# A validation and acceptability study of cognitive testing using switch and eye-gaze control technologies for children with motor and speech impairments: A protocol paper

**DOI:** 10.3389/fpsyg.2022.991000

**Published:** 2022-09-26

**Authors:** Petra Karlsson, Ingrid Honan, Seth Warschausky, Jacqueline N. Kaufman, Georgina Henry, Candice Stephenson, Annabel Webb, Alistair McEwan, Nadia Badawi

**Affiliations:** ^1^Cerebral Palsy Alliance, Discipline of Child and Adolescent Health, The University of Sydney, Sydney, NSW, Australia; ^2^Department of Physical Medicine and Rehabilitation, University of Michigan, Ann Arbor, MI, United States; ^3^School of Biomedical Engineering, The University of Sydney, Sydney, NSW, Australia; ^4^Grace Centre for Newborn Care, The Children’s Hospital at Westmead, Discipline of Child and Adolescent Health, The University of Sydney, Sydney, NSW, Australia

**Keywords:** cerebral palsy, assistive technology, cognition, assessment, disability

## Abstract

Despite the importance of knowing the cognitive capabilities of children with neurodevelopmental conditions, less than one-third of children with cerebral palsy participate in standardized assessments. Globally, approximately 50% of people with cerebral palsy have an intellectual disability and there is significant risk for domain-specific cognitive impairments for the majority of people with cerebral palsy. However, standardized cognitive assessment tools are not accessible to many children with cerebral palsy, as they require manual manipulation of objects, verbal response and/or speeded response. As such, standardised assessment may result in an underestimation of abilities for children with significant motor and/or speech impairment. The overall aim of the project is to examine and compare the psychometric properties of standardised cognitive assessment tools that have been accommodated for use with either a switch device or eye-gaze control technologies, with the specific aims to: (1) Examine the psychometric properties (measurement agreement and validity) of accommodated assessment tools by comparing the performance of typically developing children on six cognitive assessment tools administered *via* standardised versus accommodated (switch or eye-gaze control) administration; (2) Describe and compare the performance and user experience of children with cerebral palsy on six accommodated cognitive assessments administered *via* switch or eye-gaze control technologies. Secondary aims are to: (1) Describe the completion rates and time to complete assessments of participants in each group; (2) Within the group with cerebral palsy, examine the effects of condition-specific characteristics (type of cerebral palsy, functional levels, and pain) and demographics (age, socio-demographic) on participation. This protocol paper describes a two-phase validation and acceptability study that utilizes a mixed-model design. This study will collect concurrent data from 80 typically developing children and 40 children with cerebral palsy, who use switch or eye-gaze control technology as alternate access communication methods. The set of instruments will measure receptive vocabulary, fluid reasoning, sustained attention, vision perception, visuospatial working memory and executive functions. Data analyses will be conducted using SPSS v. 25 and R v 4.1.0. SPSS Sample Power 3 was used for power computation and allows for a 10% drop out rate. Quantitative descriptive statistics, measurement agreement data plotting, bivariate and multiple regressions analysis will be conducted using appropriate methods.

## Introduction

Cerebral palsy is primarily a motor disorder that entails significant risk for a range of associated impairments including behavioural difficulties, intellectual disability, and hearing, vision, and speech impairments (Rosenbaum et al., 2007). A systematic review of available international population-based data found that one in three children with cerebral palsy have a severe gross motor impairment, one in four cannot talk, one in two have an intellectual disability and one in four have a behaviour disorder (Novak et al., 2012). A population based Swedish study reported that one in two children with cerebral palsy have a speech disorder (Nordberg et al., 2013). The speech and motor difficulties associated with cerebral palsy may mask recognition of a child’s cognitive capabilities, in part due to presumptions of low capability associated with physical disability. Findings show that severity of motor impairment of children with spastic cerebral palsy correlate with severity of cognitive impairment. In contrast, for children with dyskinetic cerebral palsy, severe motor impairment and reduced language skills due to dysarthria can result in an underestimation of the child’s cognitive abilities (Fennell and Dikel, 2001). These presumptions of lower capacity may restrict access to opportunities for learning and development, as family, peers, educators and health professionals fail to recognise and respond to the child’s intrinsic developmental potential (Ashwal et al., 2004; Blair, 2016). Therefore, early detection of cognitive capability and implementation of necessary interventions are essential to optimizing the development, growth and quality of life of children with cerebral palsy; enabling them to reach their full potential (Ashwal et al., 2004; Warschausky et al., 2012). Despite the importance of knowing a child’s level of cognitive functioning, less than one-third of children with cerebral palsy have standardized assessments (Andersen et al., 2008; Sigurdardottir et al., 2008; Romeo et al., 2011; Sherwell et al., 2014; Stadskleiv et al., 2015).Currently, standardised cognitive assessment tools remain inaccessible to children cerebral palsy with significant motor and speech impairments. The most commonly used intelligence tests require quick manual manipulation of objects and/or verbal responses, which are scored according to the speed at which they are performed (Coceski et al., 2021). As such, normed comparisons of cognitive measures for children with significant motor and/or speech impairment are unlikely to yield valid estimates of cognitive capacity, particularly for children who are unable to manipulate objects and/or use assistive technology to access computers and communication devices (Miller and Rosenfeld, 1952; Hohman, 1953).

Assistive technologies can bridge the gap between physical impairments and task requirements for children with cerebral palsy who have significant motor and speech impairments (Murchland and Parkyn, 2010). Assistive technologies for communication include low-tech devices, such as picture boards and exchange systems, and high-tech devices, such as computers or electronic devices which produce synthetic or digitalised speech (Bailey et al., 2006). Mechanical switches and eye-gaze control technology are commonly used to access these high-tech devices, replacing the need to use a computer keyboard or mouse. Mechanical switches can enable the child to serially scan response options on a screen (i.e., multiple-choice scanning). Similarly, by enabling a child to control the cursor with their eyes, eye-gaze technology allows the user to make a selection onscreen by sustaining gaze on a desired response option (i.e., point and click approach). Therefore, undertaking cognitive assessment using these common communication access methods should provide more accurate information regarding a child’s cognitive capabilities, as they bypass sophisticated motor and speech requirements (Karlsson et al., 2022). Conversely, the introduction of assistive technology management may involve cognitive demands or mental workloads which are not specific to the construct of assessment interest and may prove a liability.

Enthusiasm for accessible accommodations is tempered by evidence that modifying standardized procedures to make them accessible may change the psychometric properties of tests (Hill-Briggs et al., 2007; Warschausky et al., 2012). Therefore, the Standards for Psychological and Educational Testing (American Educational Research Association (AERA), 2014) include recommendations to demonstrate comparability by pilot testing accommodations prior to clinical use and providing psychometric data regarding test validity. A systematic approach can include first examining the effects of accommodations on the psychometric properties in a sample of children who do not have significant impairments and can participate in the standard and modified versions of tests. Following demonstration of measurement agreement in a typically developing sample, subsequent research can examine the psychometric effects of accommodations in the target population.

Research has shown that it is possible to make accommodations to testing administration procedures of language assessments without negatively impacting test fidelity (Kurmanaviciute and Stadskleiv, 2017). A study by Warschausky et al. (2012) found that assessment tools with 4-item forced choice responses, such as the Peabody Picture Vocabulary Test (PPVT-III) and the Peabody Individual Achievement Test–Revised Reading Comprehension could be administered *via* switch and head mouse assistive technologies, without significantly altering the psychometric properties of the assessment tool (Warschausky et al., 2012). Event Related Potential (ERP), and other uses of electroencephalogram (EEG), is novel accommodations explored in the literature as alternate response formats and access methods for cognitive assessment for children and adults with impaired speech or fine motor skills (Alcaide-Aguirre et al., 2017; Berchio and Micali, 2022). While these studies show promise, translation of ERPs into clinical practice poses challenges, with the technology not fully ready for commercial use, and as such implementation does not appear imminent. Furthermore, the use of EEG for cognitive assessment will require significant training of qualified psychologists and allied health professionals, or development of a new work force skilled in basic cognitive assessment and ERP-facilitated testing.

Researchers have also investigated non-standardised response modalities such as finger pointing, eye-gaze and partner assisted scanning, as accommodations to increase accessibility of assessment measures (Kurmanaviciute and Stadskleiv, 2017; Fiske et al., 2020). For example, in a sample of 27 typically developing children aged 5 to 6 years old, Kurmanaviciute and Stadskleiv (2017) assessed the impact of altered response modality on measures of verbal comprehension and non-verbal reasoning. Results demonstrated that response modality did not impact performance, though partner assisted scanning was more time-consuming.

Similarly, young children’s performance on the Computer-Based Instrument for Low motor Language Testing (C-BiLLT), a test developed to assess spoken language comprehension in unintelligible or non-speaking children with motoric impairment (Geytenbeek et al., 2014), was found to be equally reliable regardless of whether eye-gaze pointing or finger pointing was used (Stadskleiv, 2020). This access method has been successfully used in a number of key research studies to date (Stadskleiv et al., 2017, 2018).

Thus, studies have demonstrated the feasibility of making accommodations to language assessments, enabling reliable assessment of receptive vocabulary. However, there is a paucity of reliable assessment tools for other domains of cognitive functioning for children with fine motor and/or speech impairments. Hence, the primary aim of this project is to examine and compare the psychometric properties of six domain specific cognitive assessment tools (receptive vocabulary, fluid reasoning, sustained attention, strategic planning, working memory, and visual perception) accommodated for use with assistive technology.

### Primary aims and hypotheses

Aim 1. Examine the psychometric properties (measurement agreement and validity) of accommodated assessment tools by comparing the performance of typically developing children on six cognitive assessment tools administered *via* standardised versus accommodated (switch or eye-gaze control) administration.

*Hypothesis 1-1*: The two types of accommodated instruments (switch or eye-gaze control) will yield standard scores that are not statistically significantly different from standardized administration counterparts.

*Hypothesis 1-2*: Accommodated instruments will demonstrate intraclass correlation indexes of agreement with standard counterparts that would be at least 0.75 (Lee et al., 1989).

*Hypothesis 1-3*: Bland Altman plots will provide further evidence of measurement agreement and test bias, with *a priori* criteria of an acceptable upper 95% confidence limit and lower 95% confidence limit of 1.96 SD of the differences between the methods as equal to or smaller than the normative standard deviation (SD = 15; Bland and Altman, 1986).

Aim 2. Describe and compare the performance and user experience of children with cerebral palsy on six accommodated cognitive assessments administered *via* switch or eye-gaze control technologies.

*Hypothesis 2-1*: Group differences (typically developing, cerebral palsy) in the nomothetic span of test scores computed as within-group bivariate correlation matrices, will not be statistically significant.

*Hypothesis 2-2*: Within the group with cerebral palsy, the access method (switch, eye-gaze) will not result in significant differences in the nomothetic spans of test scores.

*Hypothesis 2-3*: Within the group with cerebral palsy, access method (switch, eye-gaze) will not have a significant effect on user experience as indicated by personal ratings of task difficulty and satisfaction.

### Secondary aims

- Describe the completion rates and time to complete assessments of participants in each group.
- Within the group with cerebral palsy, examine the effects of condition-specific characteristics (type of cerebral palsy, functional levels, and pain) and demographics (age, socio-demographic) on participation.

## Materials and analysis

**Figure 1:**
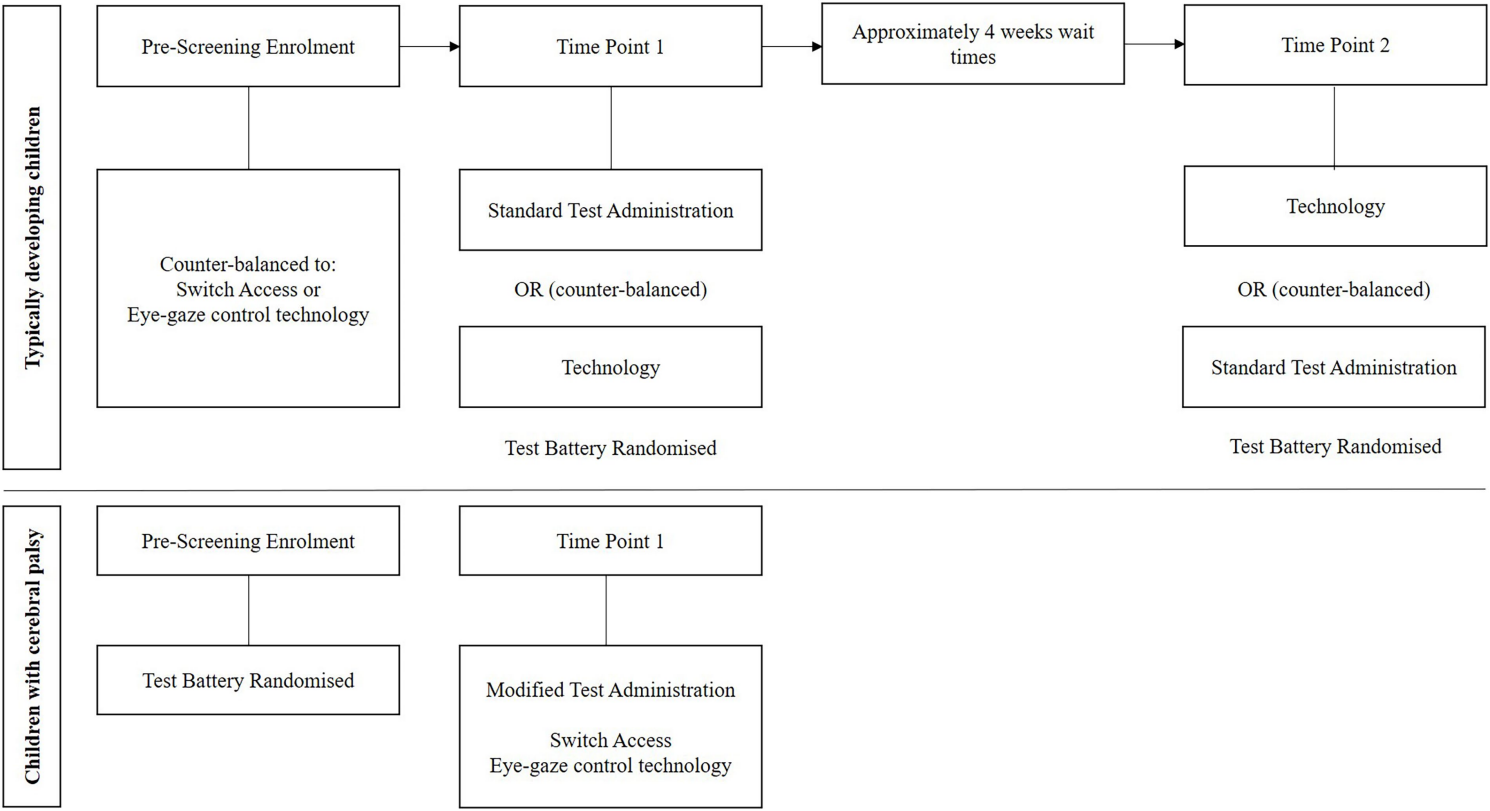
Study design.

This study is a two-phase validation and acceptability study. It adopts a mixed-model (between and within-group), mixed method (quantitative, qualitative) design to examine the utility of accommodated cognitive assessment tools for children who use switch or eye-gaze control technologies (Figure 1).

**Figure 2:**
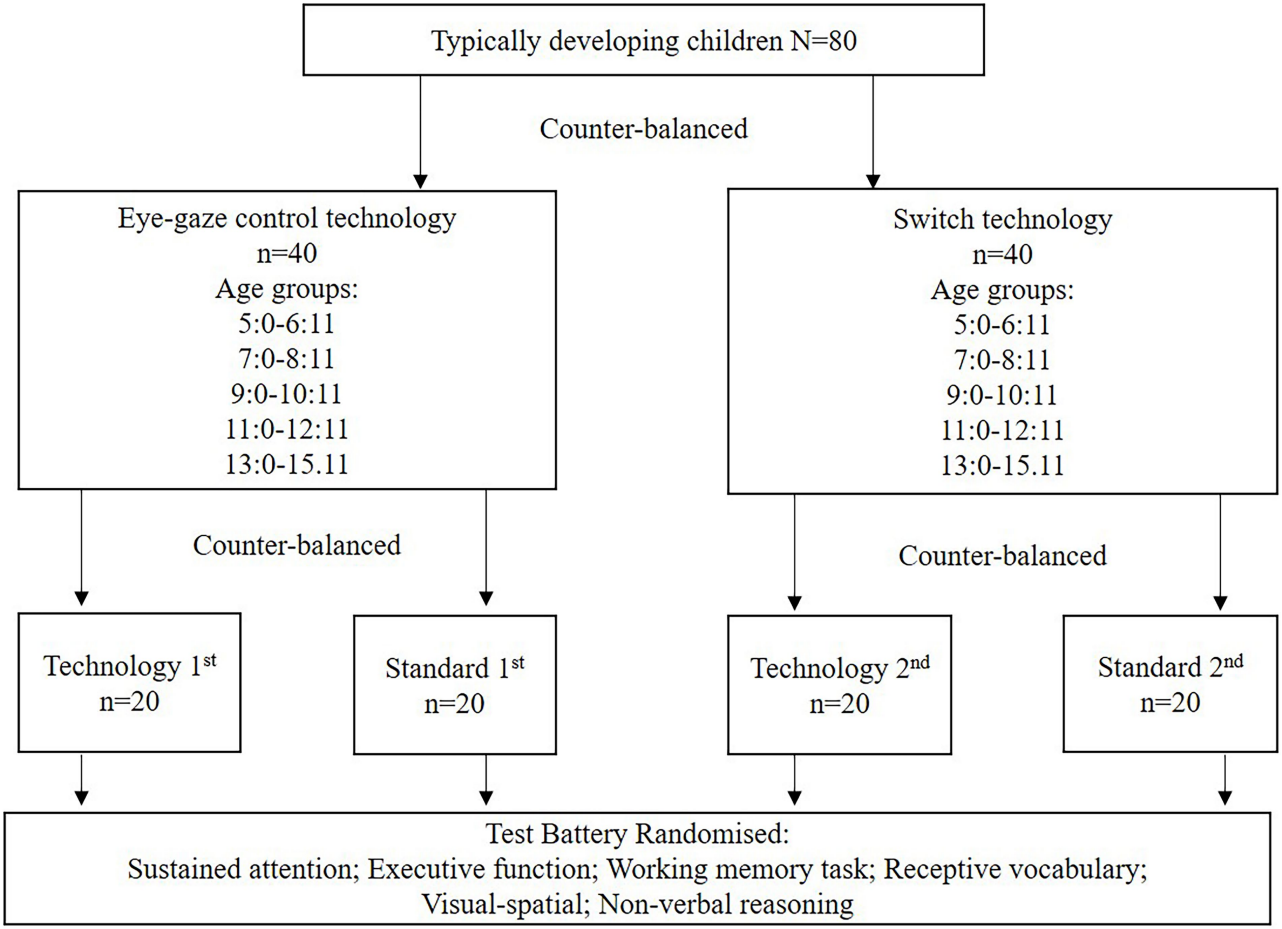
Counter balance design for typically developing children.

For typically developing children, two level counter balanced assignment will occur: (i) switch or eye-gaze control technology accommodated administration, and (ii) standard or adapted administration in the first testing session. Children with cerebral palsy will complete the accommodated assessment tools with their preferred technology, with final recruitment of equal numbers of children who use switch and eye-gaze technologies. Children with cerebral palsy will be matched at a 1:2 rate to typically developing children based on gender, Socio-Economic Indexes for Areas (SEIFA) quartiles and age. Assessment tool order will be randomized to counter potential fatigue. Assessments will be undertaken at a clinical assessment site or as a home visit (Figure 2).

### Sample size calculations

With the proposed sample size of 80 typically developing children and 38 children with cerebral palsy the study will have power of 80% at 5% overall significance to identify equivalence between the assessment administration methods for all six cognitive assessments, allowing for a 10% drop out rate in the group with cerebral palsy who may require more than one assessment session. This calculation assumes an acceptable difference between standard and modified administration methods of up to 1.25 standard deviations. This sample size will give the study power to correctly detect statistical equivalence between the administration methods in the presence of up to 0.4 standard deviations of variation between standard and modified administration scores on average, which may arise due to a learning effect from repeated assessments or the unfamiliarity of typically developing children with eye gaze or switch technology. Statistical Package Social Science (SPSS v.25) (Statistical Package Social Science, S.F.W, 2018) Sample Power 3 and R (v 4.1.0) (R Core Team, 2021) were used for the power computation.

### Eligibility criteria

#### Inclusion criteria

- Aged 5 years to 15 years 11 months
- Reside in Australian Capital Territory or New South Wales, Australia
- Comprehend one stage instructions as assessed by completion of dichotomous choice screen
- A reliable yes/no response
- Adequate hearing and vision (adjustments accepted) as assessed with caregiver screening survey and ability to pass the Dichotomous Choice Screening (Van Tubbergen et al., 2008)
- English as a primary language

#### Exclusion criteria

- Changes to medication in past month that could affect cognitive function
- Known seizure activity within the past week
- Photosensitive epilepsy
- History of psychosis
- An acute psychiatric/mental health issue of parent/guardian
- Completion of any of the included assessment tools in the past 3 months
- History of Traumatic Brain Injury
- Significant health or behavioural changes within the test–retest period

#### Additional inclusion criteria

##### Children with cerebral palsy

- Confirmed diagnosis of cerebral palsy
- Current reliance on and adequate use of switch or eye-gaze control technologies
- Able to demonstrate scrolling between screens and making selections using their preferred access method (switch or eye-gaze control technology)

#### Additional exclusion criteria

##### Typically developing children

- Impairments in dexterity and/or speech that preclude participation in standardized administrations
- Preterm birth/very low birth weight (exclusion if <37 weeks gestation or < 1500gm)
- Diagnosis of cerebral palsy or other neurodevelopmental conditions that adversely affect development
- Unable to learn to use switch or eye-gaze control technologies with training

### Recruitment

Convenience sampling using a snowball recruitment strategy will be adopted to obtain final samples of 80 typically developing children and 40 children with cerebral palsy, including 20 who use switch access and 20 who use eye gaze technology. Community awareness will be generated through flyers and correspondence with churches, sports clubs, independent/catholic schools, the Home Education Association and the NSW/ACT Cerebral Palsy Register. Personal communication with Cerebral Palsy Alliance staff, community links and campaigns on social media moderated by Cerebral Palsy Alliance will also be used for recruitment.

#### Approach to provision of information to participants and/or consent

Parents and caregivers of participating children will provide informed consent. Assent to participate in the study will always be obtained in person from any child or youth as the active participation of the person is required. Assent information has been accommodated on screen to provide children with cerebral palsy the opportunity to independently provide assent using their switch or eye-gaze control technology to let us know if they want to participate or not. Capacity to comprehend and respond to single step questions will be assessed by completion of dichotomous choice screen. This will provide a clear indication if the participant has a reliable yes/no response. Study information and assent questions will be provided in English at an early primary school level to support comprehension. Whilst it will not be possible to definitively assess comprehension of information, the assent activity will allow the participant to be familiar with the screen, demonstrate their ability to scroll between screens and make selections using their preferred access method (switch or eye-gaze control technology). It will also provide an opportunity for further discussion around what the study involves. Children who provide assent will also require parental/caregiver consent to participate in the study. Children who decline participation in the study during the assent process will discontinue involvement regardless of parental consent. If a child is unable to undertake the assent process, parental/caregiver consent and implicit child assent will be used to guide participant involvement. Implicit assent here refers to behavioural compliance and willingness undertake assessment tasks.

### Assessment procedure

After providing assent and informed consent, participants and their parent/caregiver will be invited to attend a site at Cerebral Palsy Alliance, a state-wide disability organisation. Typically developing children will attend two testing sessions where they will complete standard (paper and pencil) and accommodated administration of the assessments using switch or eye-gaze control technologies (Figure 1). Assessment will be undertaken 2–4 weeks apart and counter-balanced to reduce the impact of potential learning effects. Selection criteria and demographic information that can be obtained over the phone prior to the face-to-face appointment, will be collected. For children with cerebral palsy, condition related information and proficiency in switch and eye-gaze control technology access will also be collected. Included in the face-to-face assessment, participants will be asked to demonstrate that they can count from 1 to 20, complete a dichotomous choice test to ensure they have a reliable yes/no response, and will complete (with parent/carer assistance if necessary) the Wong Baker FACES^®^ Pain Rating Scale Revised (FPS-R; Foundation, 2018). Children with cerebral palsy will demonstrate their ability to scroll between screens and make selections using their preferred access method (switch or eye-gaze control technology).

Participants completing the assessment on eye-gaze control technology will have their scan time individualised based on participant needs, with preference recorded. Assessment will then be undertaken. Administration order of measures will be counterbalanced across participants to adjust for fatigue related affects. Where possible, assessment will be completed in one session with breaks offered as necessary. However, if required, administration will be split across multiple sessions. Administration related information will be captured by the assessor *via* the assessor questionnaire. Following assessment, participants will be asked to complete the Quebec User Evaluation of Satisfaction with Assistive Technology (QUEST; Demers et al., 2000) and the National Aeronautics and Space Administration Task Load Index measure (NASA-TLX; Hart and Staveland, 1988) about their experience of undertaking assessment. Each session will take approximately 2 h (Table 1).

**Table 1:** Included measures/tasks per participant.

**Measures/tasks**	**Description**
**Screening, consent and baseline information**	
Phone screener	Inclusion and exclusion eligibility criteria completed over the phone
Dichotomous Choice Screen	Demonstration of a reliable yes/no response
Count 1–20	Inclusion criteria
Parent/caregiver consent	Required for minors
Child assent	Obtained *via* tablet using participant’s preferred communication method
Baseline questionnaire	Socio-demographic information, basic health and development information, and condition related information (for children with cerebral palsy)
**Neuropsychological assessment tools**	
The Peabody Picture Vocabulary Test (PPVT-5)	Receptive vocabulary
Matrix Reasoning - a subtest from: Wechsler Preschool and Primary Scale of Intelligence, Fourth Edition: Australian and New Zealand Standardised Edition (WPPSI-IV A&NZ) **OR** Wechsler Intelligence Scale for Children, Fifth Edition: Australian and New Zealand Standardised Edition (WISC-V A&NZ)	Perceptual reasoning
Barking (age 5–7 years and 11 months) **OR** Vigil (age 8–15 years and 11 months) Subtests from the Test of Everyday Attention for Children 2 (TEA-ch2)	Sustained auditory attention
Tower of Hanoi (TOH)	Executive functioning including planning and problem solving
Self-Ordered Point Task (SOPT)	Visuospatial working memory
The Motor Free Visual Perception Test-4 (MVPT-4)	Visual perception
**Other standardised measures**	
Wong Baker FACES^®^ Pain Rating Scale Revised (FPS-R)	Current pain intensity score
National Aeronautics and Space Administration Task Load Index measure (NASA-TLX)	Measure of mental, physical, temporal, and overall task difficulty
The System Usability Scale (SUS)	Measure of useability and user satisfaction
**Assessor measure**	
Assessor questionnaire	Completed for each participant by the assessor. Includes quantitative and qualitative information regarding assessment duration, perceived assessment accuracy and challenges faced during administration

### Study instruments and measures

#### Primary outcome measures

The proposed assessment tools assess the cognitive domains of receptive vocabulary, fluid reasoning, attention, executive function, working memory and visual perception. These assessment tools were selected based on best available evidence, are not timed and have features that were appropriate for modification for assistive technology access (e.g., multiple choice response options).

*The Peabody Picture Vocabulary Test*, (*PPVT-5*; Dunn, 2019) will be used to measure receptive vocabulary. It provides an estimate of verbal intelligence (Dunn, 2019) for people aged 2:6–90 years. The PPVT-5 is typically used in populations with suspected speech or reading problems, for people with English as a second language, or where verbal abilities are unknown. Whilst research has demonstrated effective modification of PPVT-III, further research investigating the validity of the revised tool in a sample of people with cerebral palsy reliant on assistive technologies is necessary (Warschausky et al., 2012). The PPVT is a highly recommended supplemental instrument in the U.S. National Institutes of Health Cerebral Palsy Common Data Elements.

*The Matrix Reasoning* subtest of the *Wechsler Preschool and Primary Scale of Intelligence*, *Fourth Edition: Australian and New Zealand Standardised Edition* (*WPPSI-IV A&NZ*; Wechsler, 2012) and the *Wechsler Intelligence Scale for Children*, *Fifth Edition: Australian and New Zealand Standardised Edition* (*WISC-V A&NZ*; Raiford et al., 2016) will be used to measure fluid reasoning. WISC-V A&NZ is an international gold standard measure of intelligence for children. The Matrix reasoning subtest of the WISC-V and WPPSI-IV specifically lends themselves to discrete choice-based accommodation that allow for switch access responses. All others require manual dexterity, timed responses, or complex verbal responses which are inappropriate for accommodations.

*The ‘Barking’* (age 5–7 years and 11 months) and *‘Vigil’* (age 8–15 years and 11 months) subtests of the *Test of Everyday Attention for Children* 2 (*TEA-ch2*; Manly et al., 1998) will be used to assess the child’s ability to sustain their attention. The TEA-ch2 is a standardised, norm referenced assessment tool to measure the attentional capacity of children aged 5-16 years. Dimensions of attention are assessed *via* a number of different subtests (Evans and Preston, 2011).

The *Tower of Hanoi* (*TOH*) will be used to assess executive functions such as problem solving, planning, set shifting and mental flexibility. Traditionally developed for adult populations with neurological impairment, the TOH has since been accommodated as a measure of executive functioning for both children and infants (Welsh et al., 1991; Klahr and Robinson. 1981). In the task, the participant is shown 2, 3 and 4 disc puzzles on 3 rods. Participants are required to re-arrange the disks so that their 3 rods look identical to the examiner’s target rods, following a set of rules and completing the task in as few moves as possible (Welsh et al., 1991). The psychometric properties of the TOH have been examined extensively, with various target displays and scoring rubrics reported. Whilst reported psychometric properties vary considerably across studies (Bishop et al., 2001), it is generally considered an acceptable measure of executive functioning for children (Bull et al., 2004; Anderson et al., 2010).

The *Self-Ordered Point Task* (*SOPT*), developed by Petrides and Milner (1982) will be used to assess visuospatial working memory. Here, participants are shown sets of 6, 8, 10 and 12 abstract images (Strauss et al., 2006). The same images are shown over as many pages as there are images in the set, but the images change location on each page. Participants are instructed to touch a different picture on each page. There are 3 trials for each set, increasing cognitive load as participants are required to recall which pictures have been touched on each trial. Whilst a less commonly used measure of working memory, this is one of the few working memory tasks with psychometric evidence to support its use (Ross et al., 2007), which can be accommodated for eye-gaze and switch access technologies.; results are not time dependent.

The *Motor Free Visual Perception Test-4* (*MVPT-4*; Colarusso and Hammill, 2015) will be used to assess visual perception. The MVPT-4 is a standardised norm-referenced assessment tool for children and adults aged 4 years or older (Colarusso and Hammill, 2015). As a forced choice matching task, the MVPT-4 does not require motor responses, and is appropriate for accommodations for children who use eye-gaze and/or switch access technology to communicate. The normative data is reportedly representative of the US population, and it has shown good content and construct validity (Brown and Peres, 2018) and excellent test–retest reliability (Chiu et al., 2022).

Permission to make accessibility accomodations to copyrighted measures, for the purposes of this research project, has been sought.

#### Secondary/covariate measures

*The Wong Baker FACES® Pain Rating Scale Revised* (*FPS-R*; Wong et al., 1996) will be used to measure pain. The FPS-R is a pain measurement tool made up of 6 pictures of faces numbered 0–10 ranging from “no pain” to “worst pain imaginable.” It was originally created to help children communicate their pain and is now used internationally. Permission to use the FPS-R for this project has been granted.

*The National Aeronautics and Space Administration Task Load Index measure* (*NASA-TLX*; Hart and Staveland, 1988) will be used to evaluate task difficulty. The NASA-TLX measures mental, physical and temporal demand as well as performance, effort and frustration on a 21-point scale, comprised of 7 markers. Low, medium and high rankings comprise each marker.

*The System Usability Scale* (*SUS*; Lewis, 2018) will be used to evaluate user satisfaction. The questionnaire consists of 10 usability items. For each of the 10 items, the participant’s usability of the technology with the assistive device will be assessed using a 10-point scale.

*A Baseline Questionnaire* has been designed to gain some basic socio-demographic information such as age, Socio-Economic Indexes for Areas (*SEIFA*), cognitive testing in the last 12 months, and health/development related information, such as gestational age at birth, level of education, developmental difficulties, vision, and hearing ability. For children with cerebral palsy, condition specific information is also collected using: Gross Motor Function Classification System Expanded and Revised (GMFCS E&R; Palisano et al., 2007), Manual Ability Classification System (MACS; Eliasson et al., 2006), Communication Function Classification System (CFCS; Hidecker n.d.) Viking Speech Scale (VSS; Pennington et al., 2013), and Eating and Drinking Ability Classification System (EDACS; Sellers et al., 2014). Type of computer access method (switch or eye-gaze control technology), individual technology settings, and how long they have used the technology will also be recorded for children with cerebral palsy.

*An Administrator questionnaire* has been designed to gather a description from the assessor on their subjective experience of further improvements in regards to the technical set up and accessibility of the assessment battery for the individual participant. The assessor will also record the number of sessions it took to administer the assessment battery and assessment completion time for each participant.

### Equipment

**Figure 3:**
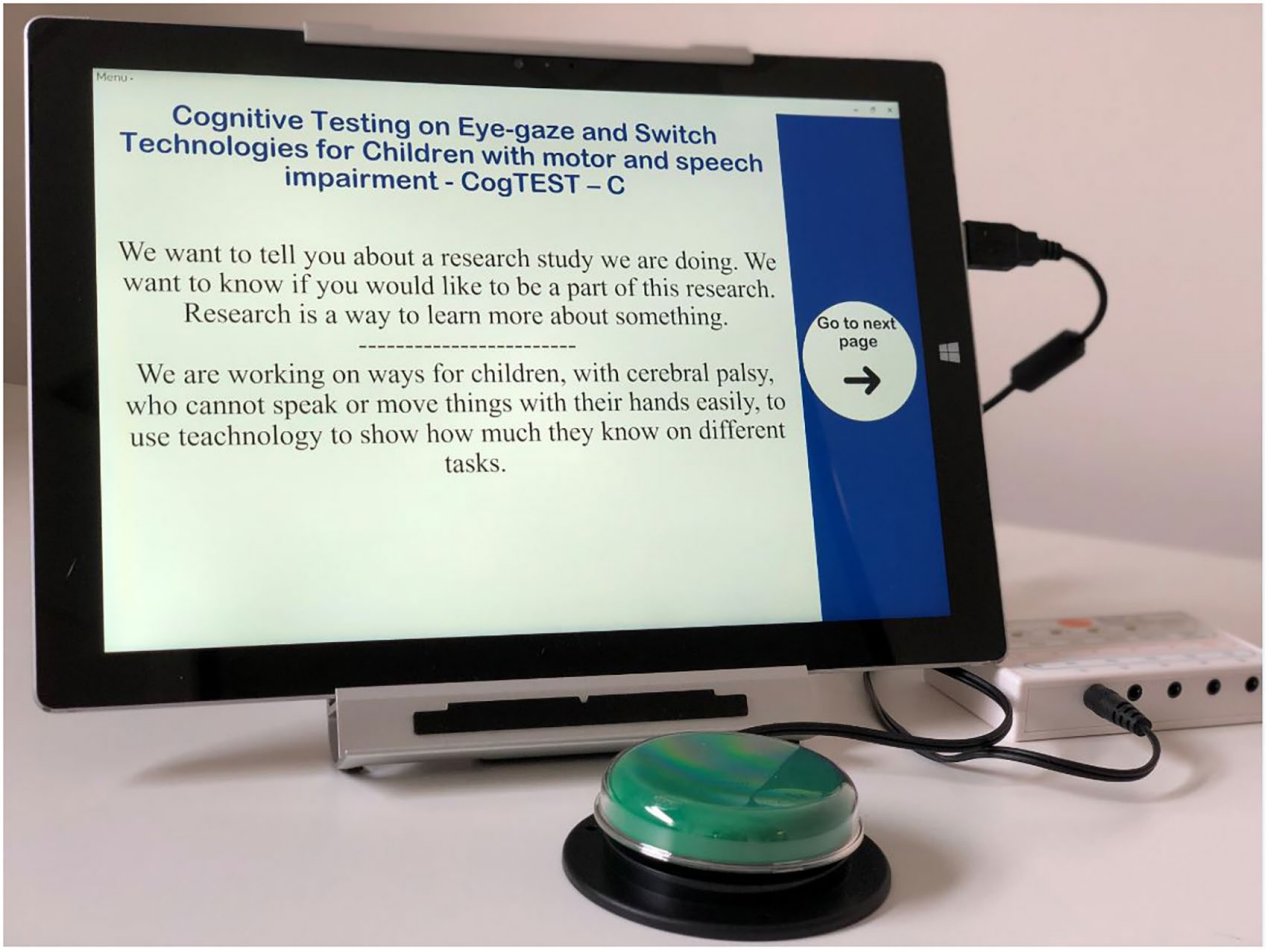
Switch assess.

*Switch access* is an assistive technology which replaces the need to use a computer keyboard or a mouse. The switch can be utilised with a row-column presentation format for forced choice (Figure 3).

**Figure 4:**
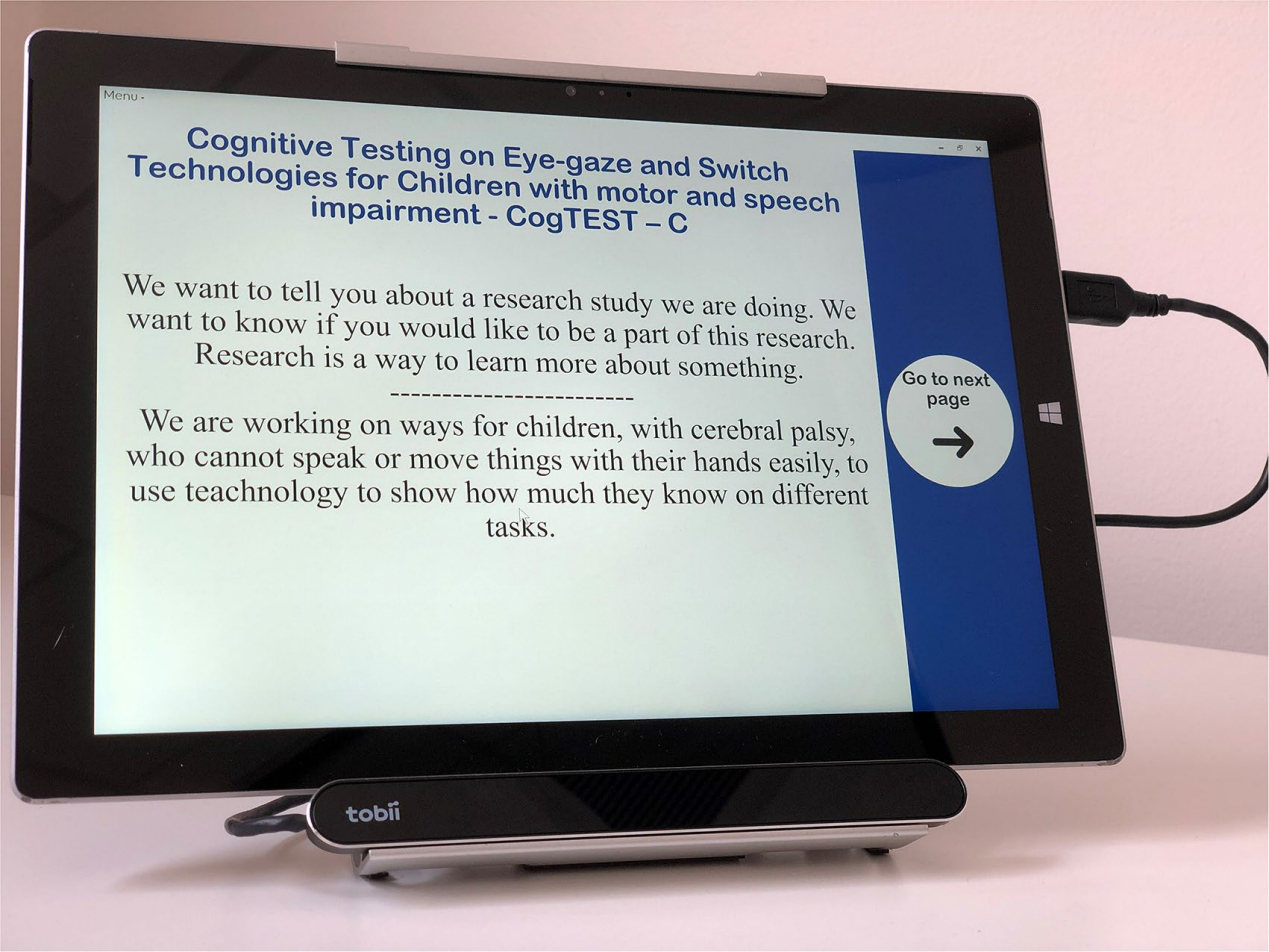
Eye-gaze control technology.

*Tobii PCEye mini eye-gaze control technology* involves a specialised video camera mounted below the tablet PC which tracks the user’s eyes. Sophisticated image-processing software in the tablet continually analyses the camera’s image of the eye and determines where the user is looking on the screen (Tobiidynavox, 2016). To select a target the person holds their eye-gaze for a certain time, referred to as ‘dwell’ (Figure 4). If a participant with cerebral palsy is used to a different brand of eye-gaze control technology device, and can be moved across to the study computer, that device can be used if that is the choice of the participant, as there are subtle differences between devices and theirs might be the best fit for their gaze control.

To enable our assessment tools to be switch and eye-gaze control technology accessible on a Window based platform, five of the six assessment tools were accommodated using Grid 3 software (Thinksmartbox, 2018). The tablet to be used in this study will be a Surface Pro 4.29 × 20 cm. Participants will sit approximately 80 cm from the screen. Standard administration of TEA-Ch-2 does not require accommodation. However, a page in Grid 3 was developed with numbers 1–20 randomly placed on the display for the participant to provide the response. The screen will not be displayed until after each trial.

Grid 3 is a software solution which is designed for people with complex communication and or access needs. It includes features which enables the user to control the computer and their environment with a switch, eye-gaze control technology and other assistive technology access solutions.

The assessment tools were accommodated to display the test items as they would be presented in the paper version according to each test manual. However, three communication buttons will also be presented on each screen. This will enable participants to confirm their response selection, or let the test administrator know if they accidently select the wrong answer (perhaps due to eye-gaze or switch control anomalies) or are unsure about the selection. This additional confirmation step is necessary to remove the likelihood of false negatives and positives, and allow some qualitative communication throughout assessment to reduce frustration and maintain rapport. Two additional buttons to navigate to the next and previous page will also be available at the bottom of the screen and will only be accessible to the administrator.

### Data processing and analysis

Data analysis will be conducted using SPSS v. 25 (Statistical Package Social Science, S.F.W, 2018) and R v 4.1.0 (R Core Team, 2021). Raw scores on standardised assessment tools will be converted to scaled scores and/or standard scores where appropriate. Raw scores on the TOH and SOPT will be converted to z-scores using available published data. Descriptive statistics will be used to summarise demographic and clinical characteristics, assessment tool performance, secondary outcomes, and user experience. Prior to undertaking any statistical analysis, the normality of the scaled/standardised scores and Z-scores for the six assessments tool will be assessed overall and for each combination of administration and access method (i.e., standard administration and switch; standard administration and eye gaze control technology; person with cerebral palsy using switch; and person with cerebral palsy using eye-gaze control technology) using Shapiro-Wilks assessment and visual inspection of histograms.

The measurement agreement and validity of the accommodated assessment tools will be investigated by testing hypotheses of equivalence between scores from the standard administration and scores from the accommodated administration of the assessments. These hypotheses will be tested using paired t-tests for Wilcoxon signed-rank tests if the data is not normally distributed. The significance level for these tests will be adjusted to account for multiplicity and control the overall Type I error rate at 5%. Correlations will be investigated between performances on each of the six assessments for all combinations of access methods, and intraclass correlation coefficients (ICC) will be calculated. For the sample of children without cerebral palsy, the sample size will be sufficient to examine Bland Altman plots for further evidence of measurement agreement and test bias. In addition, we will compare the bivariate correlation matrices with adaptive test scores to examine the nomothetic spans of instruments by comparing coefficients using Fisher’s r to z transformations. Regression models will be investigated to examine between group differences in (i) children with cerebral palsy using switch and eye-gaze control technologies for each of the six assessment tools and for the two user experience measures, and (ii) between typically developing children’s and children with cerebral palsy’s performance using switch and eye-gaze control technologies, across all six assessment tools. Potential confounders and effect modifiers will be investigated for these regression models Associations between test results and secondary outcomes for the children with cerebral palsy will be investigated using chi-squared or Fisher’s exact tests.

## Discussion

Many cognitive assessment tools are inaccessible for children with significant motor and/or speech impairments. This is in violation of the basic human right of equality of access to health care. As a result, children with significant physical disability who also require support with cognitive development may not receive timely early intervention, nor appropriate supports and opportunities in the school environment. It is equally important to identify people with severe cerebral palsy who have normal to high levels of intelligence to allow them to achieve their educational and employment potential. Clearly, accurate assessment of cognitive capabilities will also facilitate optimal participation of the individual in medical decision-making. This is fundamental to self-determination, in accordance with the United Nations Convention on the Rights of Persons with Disabilities.

This study’s overall aim is to examine the psychometric properties of six cognitive assessment tools when accommodated for use with switch and eye-gaze control technologies. To enable our assessment tools to be switch and eye-gaze control technology accessible on a window based platform, all of the six assessment tools were accommodated using Grid 3 software (Thinksmartbox, 2018).

To our knowledge, no study has yet been carried out with the number of participants required to adequately power an investigation of accessible accommodations to a range of cognitive assessment tools. As standardised administration procedures require pointing or verbal responses, administration using these procedures would be unethical and infeasible for the children with cerebral palsy included in this study, who by selection are unable to point or provide verbal responses.

As many of the proposed participants will likely have never been exposed to a test environment, or used their assistive technology to complete cognitive assessments, they may experience fatigue, or not fully comprehend the expectation of a test/examination context. If this occurs, the assessment tools may be administered over two sessions, rather than the single session necessary for neurotypical peers. Generous time for the testing of assessment tools in children with cerebral palsy has been scheduled as they may need more practice and time to settle into the tests.

There are innovative aspects of this research. First, the participant information and assent material has been accommodated to provide children with cerebral palsy the opportunity to independently provide assent through the use of their switch or eye-gaze control technology. To our knowledge this is the first time this has been designed in an adequately powered study. The protocol states that if a participant indicates that they do not wish to participate even if their caregiver has provided consent, the wish of the child will be respected and the participant will not be exposed to the research activity. Moreover, this is the first time a comprehensive standardised assessment battery will be made accessible and administered to children with significant motor and/or speech impairment without any content modifications. Finally, a knowledge translation strategy will be developed by the investigator and consumer advisors panel to target, in the most appropriate ways, key stakeholders to ensure that research findings can be rapidly and effectively translated into practice and into improving outcomes for consumers.

Having access to communication is a human right. We must use technology to break down accessibility barriers and provide children with cerebral palsy modified assessments. Only then we can gain an informed understanding of their cognitive and receptive language strengths and weaknesses. This information will not only provide the foundation for right interventions but also the right opportunities for children with cerebral palsy.

## Author’s note

The study findings will be disseminated through peer-reviewed publications, international conferences and through the consumer advisory panel in the study.

## Ethics statement

This study was approved by the University of Sydney, Human Research Ethics Committee, Sydney, Australia, reference no. 2020/469. Written informed consent to participate in this study will be provided by the participants and the participants’ legal guardian/next of kin.

## Author contributions

PK and IH conceived the study concept, developed the funding proposal, applied for funding and initiated the writing of the protocol paper, and will coordinate the project. CS will conduct the study. GH will provide administrative support throughout the project. AW developed the statistical analysis plan and will contribute with the interpretation of the data. SW and JK provided expert opinion in administration of accommodated cognitive assessment tools. AM provided expert opinion in technology aspects of the protocol. NB provided expert opinion in cerebral palsy and child development. PK is the chief investigator. IH, NB, and PK are the grant holders for the technology development and accommodations within this study. All authors contributed to the article and approved the submitted version.

## Funding

The following authors’ participation will be partly supported by the Roger Montgomery Family Trust (PK), the Neurodisability Assist Trust (IH), and the Sydney Medical School Foundation (NB).

## Conflict of interest

The authors declare that the research will be conducted in the absence of any commercial or financial relationships that could be construed as a potential conflict of interest.

## Publisher’s note

All claims expressed in this article are solely those of the authors and do not necessarily represent those of their affiliated organizations, or those of the publisher, the editors and the reviewers. Any product that may be evaluated in this article, or claim that may be made by its manufacturer, is not guaranteed or endorsed by the publisher.
